# A Dual‐Channel Synergistic Ultrasensitive Biosensor for Tumor Liquid Biopsy

**DOI:** 10.1002/advs.75443

**Published:** 2026-04-27

**Authors:** Yu Sun, Jing Zhang, Chenqing Liu, Jing Lou, Sihui Wang, Xiangtian Ji, Xiaofang Zhao, Shirui Xu, Jingyu Feng, Bin Liu, Chao Chang, Qinggang Ge, Jun Yang

**Affiliations:** ^1^ Department of Neurosurgery Peking University Third Hospital Beijing P. R. China; ^2^ Innovation Laboratory of Terahertz Biophysics National Innovation Institute of Defense Technology Beijing P. R. China; ^3^ Department of Otorhinolaryngology, Head and Neck Surgery The Sixth Medical Center of the Chinese PLA General Hospital Beijing P. R. China; ^4^ Department of Intensive Care Unit Peking University Third Hospital Beijing P. R. China; ^5^ Center for Precision Neurosurgery and Oncology of Peking University Health Science Center Beijing P. R. China; ^6^ Air and Missile Defense College Air Force Engineering University Xi'an P. R. China; ^7^ School of Physics Peking University Beijing P. R. China

**Keywords:** Glioma, IDH1, Liquid Biopsy, Metasurface, QBIC

## Abstract

Liquid biopsy technology, which detects circulating tumor DNA (ctDNA) in cerebrospinal fluid, plays a crucial role in the early diagnosis and precision treatment of gliomas. However, the intrinsically low abundance and rapid clearance of ctDNA pose substantial challenges for conventional analytical techniques, such as droplet digital PCR and next‐generation sequencing. Here, we introduce a label‐free dual‐channel synergistic detection strategy that enables quantitative analysis of ctDNA at the zeptomolar level. Leveraging a synergistic design that includes structural symmetry breaking and momentum condition regulation, the optical platform overcomes the inherent structural field confinement and enhances the effectiveness of target‐contacted sensing. By integrating a DNA tetrahedron‐hybridization chain reaction‐gold nanoparticle cascade amplification system, the dual‐channel synergistic biosensor achieves an ultra‐low detection limit of 74 zm while covering a dynamic range spanning 12 orders of magnitude. Clinical validation has confirmed that this dual‐channel synergistic detection strategy can accurately identify the IDH1.R132H mutation in glioma patients with as little as 1 µL of sample, and deliver crucial clinical information such as tumor size and staging. This label‐free, rapid, cost‐effective, and highly sensitive biosensing platform offers a powerful tool for early cancer screening, molecular diagnostics, and longitudinal disease monitoring.

## 1 Introduction

Glioma, one of the most common primary tumors of the central nervous system, accounts for approximately 80% of primary malignant brain tumors [1, 2]. Molecular diagnosis serves as a crucial clinical standard for its management [3, 4]. The isocitrate dehydrogenase 1 (IDH1)‐mutant glioma represents a distinct molecular subtype with high clinical relevance, characterized by a more favorable prognosis and prolonged overall survival [5, 6, 7, 8]. Unlike peripheral solid tumors, gliomas cannot be repeatedly biopsied with ease for tissue acquisition [9]. As a noninvasive approach, liquid biopsy holds great potential for early screening, precision therapy, and disease status evaluation in glioma patients [10, 11]. Due to the unique structure of the blood‐brain barrier, cerebrospinal fluid (CSF)‐derived circulating tumor DNA (ctDNA) provides a more suitable source for liquid biopsy in gliomas [12]. However, studies have shown several limitations. Gliomas of different grades indicate varying degrees of invasiveness and tumor burden, leading to substantial fluctuations in ctDNA concentration [13, 14]. Moreover, analyses of glioma‐derived ctDNA using conventional techniques such as next‐generation sequencing and droplet digital PCR (ddPCR) need improvement [15, 16]. In a cohort of 37 glioma patients, ctDNA positivity was detected in only 59% of cases [17], while another study involving 85 patients reported ctDNA detection in approximately half of the samples [18]. These suboptimal results stem from the inherently low abundance and short half‐life of ctDNA released by malignant brain tumors, which often result in frequent false negatives and diagnostic delays. Therefore, developing highly sensitive and efficient detection platforms capable of lowering the analyte response threshold and expanding the linear response range is of significant importance.

Recently, metasurfaces, with low‐profile, compact scales and highly customizable electromagnetic performance, have been integrated with rapid, high‐efficiency optical techniques to revolutionize biosensing [19, 20, 21, 22]. Through suitable material selection and elaborate structural design, a variety of exotic resonant modes with localized electromagnetic field at the micro/nanoscale strongly enhance light‐matter interaction, such as surface plasmon resonance (SPR) [23], Fano resonance [24], electromagnetically induced transparency [25], exceptional points [26], and quasi‐bound states in the continuum (Q‐BICs) [27, 28, 29]. Among these modes, the Q‐BICs excite ultrahigh quality factors (Q‐factors) due to the low‐loss characteristics of materials, enabling lab‐level biosensing applications including lung cancer cell identification [30], amino acid analysis [31], pathogen screening [32], and liquid analyte monitoring [33]. However, the theoretically weakly coupled or decoupled BIC/Q‐BIC modes and the free‐space radiation channel confine most of the electromagnetic field within the structure, thereby hindering effective monitoring of extremely low‐abundance target substances and impeding clinical application.

**FIGURE 1:**
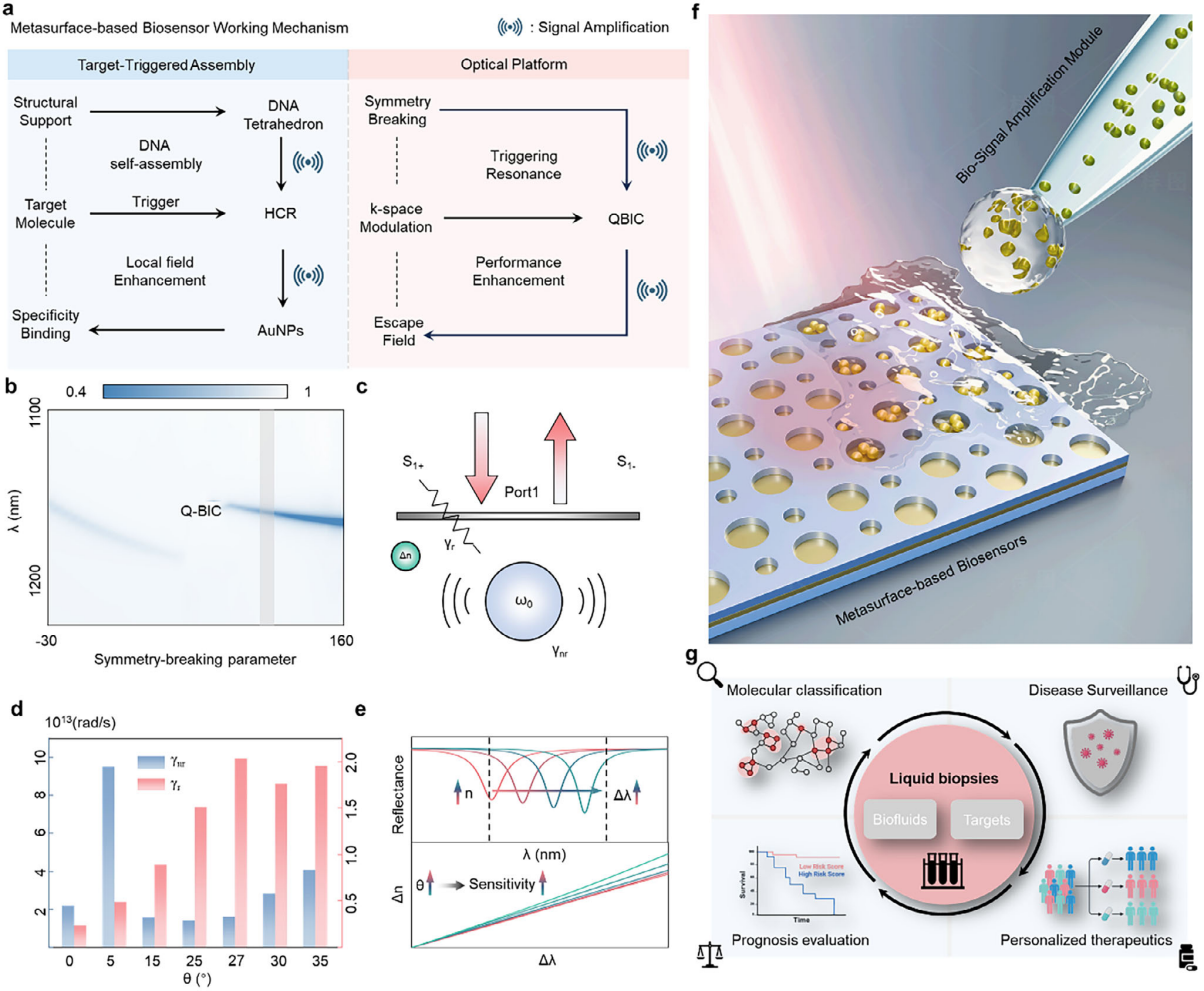
A dual‐channel synergistic paradigm for ultrasensitive ctDNA detection. (a) Label‐free dual‐channel co‐sensing strategy: (i) A self‐assembled, sequence‐specific ctDNA capture platform amplifies the biochemical signal. (ii) A dual‐channel synergistic optical platform enhances the local electromagnetic field by breaking symmetry in both structural and k‐spaces. (b) BIC resonance with enhanced intensity by increasing the symmetry‐breaking parameter, in which the symmetry‐breaking parameter is defined as the difference between the radius of circular apertures at diagonal positions (As shown in Figure 1f). (c) Schematic of Time‐Coupled Mode Theory for a single mode in a single‐port system. (d) Non‐radiative and radiative losses are modulated by changes in momentum symmetry breaking. (e) (i) Specific resonances undergo frequency shifts due to the refractive index changes in the analyte, where the color change from red to green corresponds to increasing refractive index. (ii) Sensing performance is enhanced through k‐space symmetry breaking under certain conditions, in which the transition from green to red indicates growing θ and sensitivity. (f) Schematic of DCSU‐Biosensor detection. (g) Significance of DCSU‐Biosensor in clinical liquid biopsy applications.

To address those challenges, we propose a label‐free dual‐channel co‐sensing strategy comprising a Target‐Triggered assembly and optical platform to achieve ultrasensitive diagnostics and high‐precision identification (Figure 1a). Breaking structural symmetry (The breaking parameter is selected as 100 nm, marked by a gray banded region in Figure 1b) and modifying momentum conditions, the Dual‐Channel Synergistic Ultrasensitive Biosensor (DCSU‐Biosensor) excited Q‐BIC modes with a high Q‐factor of 197. The radiative and non‐radiative losses of the DCSU‐biosensor are precisely modulated on demand (Figure 1c,d), thereby enhancing the effective field and improving sensing performance (Figure 1e). In parallel, DNA tetrahedral nanostructures (TDNs) are employed as nanoscale scaffolds, utilizing hybridization chain reaction (HCR) to achieve target‐induced signal amplification. Gold nanoparticles (AuNPs) are further incorporated to enable cascading optical signal enhancement (Figure 1f). Experimental results show that this label‐free dual‐channel co‐sensing strategy can achieve an ultra‐low detection limit of 74 zM and a dynamic range spanning 12 orders of magnitude. Clinical validation shows accurate identification of IDH1‐mutant gliomas, even in 1 µL samples, and correlation with key clinical information, including tumor size and stage. Moreover, the DCSU‐Biosensors offers higher sensitivity (93.33%) and efficiency (1–1.5 h) than ddPCR. We believe this miniature, label‐free, and highly sensitive biosensing platform establishes a new paradigm for compact, cost‐effective, and high‐performance biomolecular detection, demonstrating significant potential for liquid biopsy in tumors (Figure 1g).

## 2 Results and Discussion

### 2.1 Dual‐Channel Synergistically Induced Q‐BIC With Efficient Field‐Enhanced for Biosensor

**FIGURE 2:**
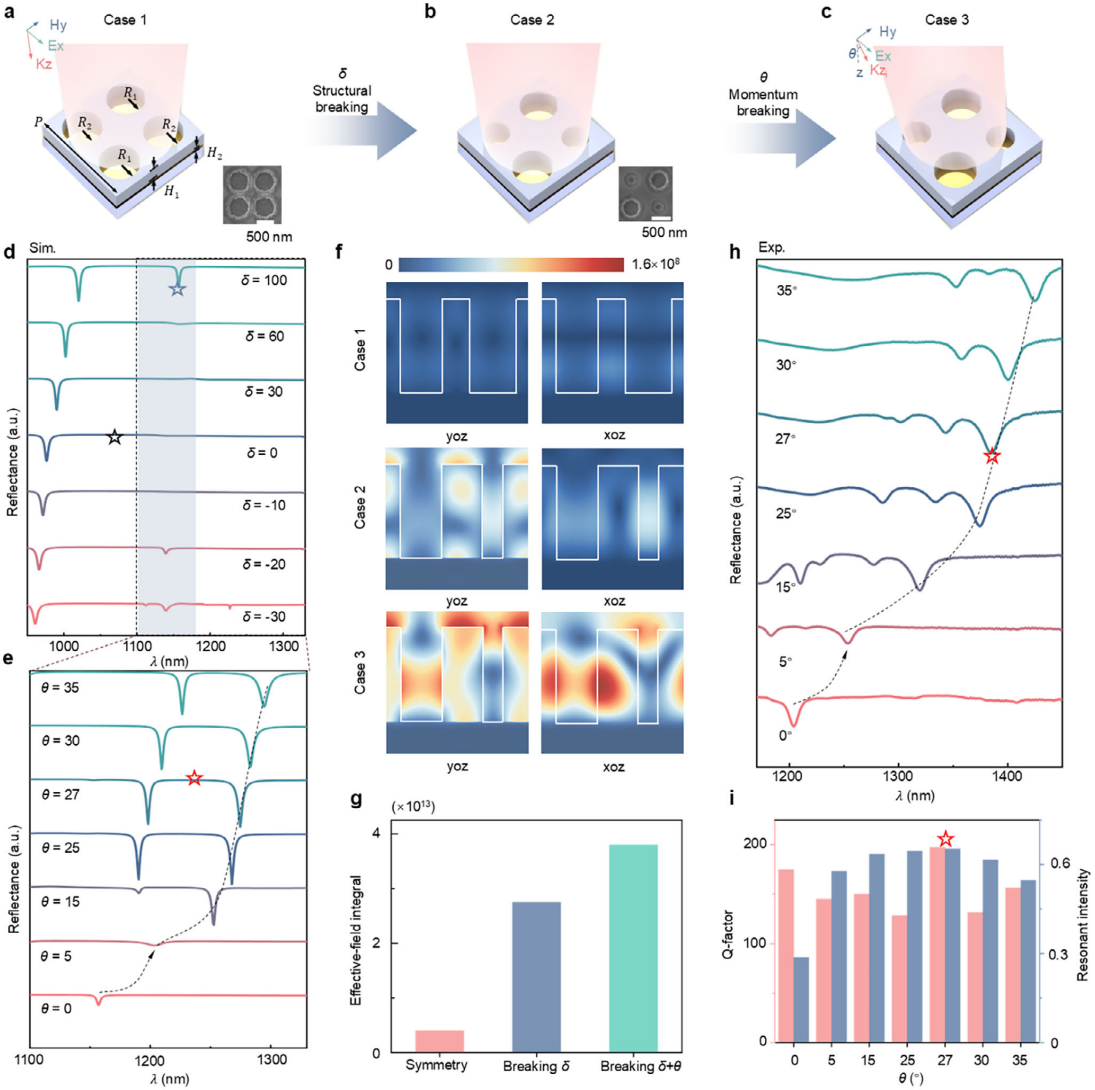
Spilled Field Enhancement Optical Platform Enabled by Structure‐k Space Symmetry Breaking. (a) Tetrameric metal‐all‐dielectric hybrid optical platform. The detailed parameters: *P* = 1400 nm, *R₁* = 200 nm, *R_2_* = 200 nm, *H₁* = 100 nm, *H_2_* = 600 nm. Inset: SEM image (scale bar: 500 nm). (b) Schematic of the optical platform with structural symmetry breaking. Inset: Corresponding SEM image. (c) A structure‐momentum co‐breaking case with enhanced spilled field for improved sensing sensitivity. (d) Simulated reflectance for various symmetry‐breaking parameters *δ*. (e) Simulated reflectance under k‐space symmetry breaking for the optical platform with *δ* = 100 nm. (f) Electric field distribution for: (i) the primitive meta‐atom, (ii) only structural symmetry breaking, and (iii) structure‐momentum space co‐breaking. (g) Integrated electric field intensity within the effective sensing region. (h) Measured reflectance spectra of fabricated DCSU‐biosensor. (i) Q‐factor and resonance strength extracted from the measured results.

To achieve a strong localized field for sensitive biosensing, we propose a metal‐dielectric hybrid optical platform. As shown in Figure 2a, the meta‐atom is sandwich structure comprising a 500 µm thick double‐polished fused silica substrate, a gold layer (*H_2_* = 100 nm, providing a reflective backplane and forming stable bonds with sulfur groups in biomolecules), and a silicon nitride layer (*H_1_* = 600 nm, reducing the effective loss coefficient to generate a strong localized field and excite a high Q‐factor resonance). Then, the circular hole geometry possessing polarization‐insensitive characteristics is selected as the base configuration to ensure device reusability and universality. Here, the tetrameric symmetry is broken to excite Q‐BIC modes, in which the parameter defined as δ = *R*_1_ − *R*_2_ is introduced here to quantify the degree of symmetry breaking (Figure 2b).

First, the reflectance at normal incidence for different δ values is examined using the Finite Element Method (simulation details are provided in Methods), and the results are presented in Figure 2d. At *δ* = 0, a resonance is observed at *λ* = 976 nm. As *|δ|* increasing, an additional resonance appears near 1156 nm (highlighted by a blue bar). The Q‐factor and resonance strength of this new resonance vary with *δ*, a characteristic feature of BIC. To enhance sensing performance while maintaining a high Q‐factor, momentum‐symmetry breaking is applied to increase the spread of localized field energy into the holes and the surrounding space (see Figure 2c). The parameter *θ* is defined as the angle between the incident light and the ‐z axis. Transmission for δ = 100 nm is shown in Figure 2e as a representative example, with θ varying. As θ increases from 0° to 5°, the resonance weakens slightly. With further increase in θ, the resonance becomes broader, with observable splitting. To understand the underlying mechanism, the multipole decomposition shows that the resonance is dominated by electric quadrupoles (Note S1 provides further details). Figure 2f displays the electric field distributions for the original configuration, the case with structural symmetry breaking only, and the case with both structural and momentum symmetry breaking. The co‐breaking case clearly allows field energy to extend into the holes and above the structure. For quantitative comparison, the integrated electric field within the holes is plotted in Figure 2g. Relative to both the original and the structurally broken cases, the structure–momentum co‐breaking increases the electric field intensity by 845% and 38%, respectively, yielding a significantly enhanced intensity.

For a deeper understanding, we employ the Time‐Coupled Mode Theory for a single‐mode in a single‐port system as follows (See Figure 1c),

$$\frac{\partial A}{\partial t}=-i\omega_{0}A-\gamma A+\kappa^{*}S_{+}$$

$$S_{-}=S_{+}+\kappa A$$

$$\gamma =\gamma_{r}+\gamma_{nr}$$

where *A* is amplitude, ω_0_ is resonant frequency, *S*_−_ and *S*_+_ is output and input of single port, and γ_*r*_and γ_*nr*_ is radiative and non‐radiative loss, respectively. By the principle of energy conservation, the reflectance could be obtained as follows [34]:

$$r\;\left(\omega \right)=-1+\frac{2\gamma_{r}}{i\left(\omega +\omega_{0}\right)+\gamma_{r}+\gamma_{nr}}$$

The fitting results are shown in Figure 1d. As the angle increases from 0° to 5°, the non‐radiative loss rises, resulting in a weakening resonance depth. Further increasing momentum breaking, the non‐radiative loss decreases while the radiative loss increases, reaching a maximum at *θ* = 27°. This confirms enhanced coupling between free space and DCSU‐Biosensor at *θ* = 27°, which allows the electric field to leak into the surrounding environment and improves the sensing performance. To validate the simulation, we fabricated the synergy‐broken optical platform and measured the corresponding reflectance. The measured result is depicted in Figure 2h, which aligns well with the simulated trend. The minor resonance wavelength shift is attributable to fabrication tolerances. Furthermore, the corresponding Q‐factor and resonance strength extracted from the experimental data are summarized in Figure 2i. Here, the Q‐factor and resonance intensity are calculated by $Q=\frac{\lambda_{0}}{\Delta \lambda }\;$ and *R_peak_* − *R_valley_*, where λ_0_, Δλ, *R_peak_* and *R_valley_* denote the resonant wavelength, half‐width, reflection peak, and reflection valley, respectively. At *θ* = 27°, the corresponding quality factor reaches 197, and the resonance intensity is 0.653.

### 2.2 Self‐Assembly of TDN–HCR–AuNPs Complexes

**FIGURE 3:**
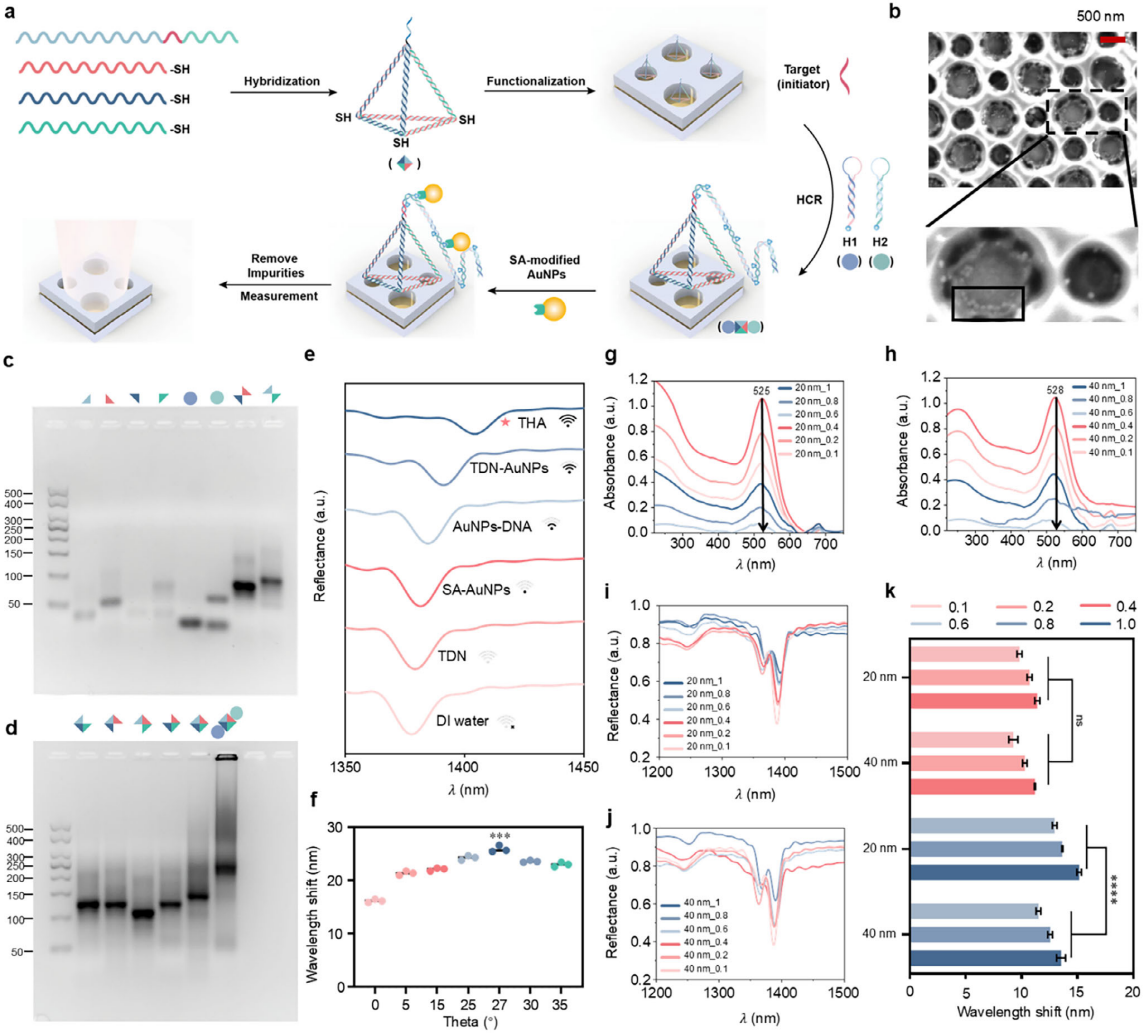
Characterization and optical performance of THA composites assembled on the DCSU‐Biosensor surface. (a) A schematic of THA composites’ self‐assembly on the device surface. (b) SEM images reveal AuNPs recruited by the self‐assembled products. (c,d) AGE analysis of self‐assembled products, with lane indicators represented by icons above each lane. (e) Spectral reflectance of self‐assembled products. (f) Wavelength shift of the THA product as a function of incident angle (*n*  =  3). (g,h) Ultraviolet–vis absorption spectra of SA‐AuNPs (20 nm and 40 nm) at varying OD values. (i,j) Reflectance spectra of 20 nm and 40 nm SA‐AuNPs at different OD values. (k) Comparison of signal enhancement effects using 20 and 40 nm SA‐AuNPs for metasurface sensing (*n*  =  3).

To further enhance the sensitivity of DCSU‐Biosensor for detecting glioma biomarkers, we developed a “TDN–HCR–AuNPs (THA)” cascade signal amplification strategy (Figure 3a). This method leverages the rigid 3D architecture of TDN to immobilize the capture probes. The CG base content in the sequence affects the synthesis efficiency of TDN (Figure S2). Based on the IDH1 exon 4 R132H mutation sequence (52 bp) as the trigger region, the probes on TDN were rationally designed. Additionally, both H1 and H2 are hairpin structures. For subsequent conjugation, biotin modification is introduced at the 5' end of H1 and the 3' end of H2. Target binding exposes the trigger region, initiating the HCR. This process involves the successive opening and hybridization of H1 and H2 hairpins, leading to the formation of extended DNA nanostructures. These elongated DNA products subsequently recruit AuNPs through biotin–streptavidin (Biotin–SA) specific interactions, markedly amplifying the optical response of ctDNA on the metasurface.

Scanning electron microscopy (SEM) observations reveal dark, punctate nanostructures with an average diameter of approximately 20 nm distributed along the pore edges and within the central regions of the substrate, confirming that the self‐assembled complex efficiently concentrates AuNPs within the substrate cavities (Figure 3b). The morphology of the THA nanostructure is further examined by transmission electron microscopy (TEM) (see Figure S3a). Furthermore, the synthesized DNA components are characterized via agarose gel electrophoresis (AGE): the four single strands constituting the TDN, along with H1 and H2, exhibit lengths ranging from 40 to 100 bp; the TDN duplex intermediates appear between 80 and 100 bp (Figure 3c), the triple‐stranded intermediates between 100 and 150 bp, and the fully assembled TDN at approximately 150 bp. The HCR products displayed electrophoretic bands concentrated at 250–300 bp (Figure 3d). Owing to the spatial folding effect of DNA molecules, slight deviations between the experimental and theoretical lengths are observed; nevertheless, the results still provide clear validation for the successful execution of HCR by TDN. Collectively, these findings demonstrate the feasibility of the proposed THA cascade signal amplification strategy.

Under optimized experimental conditions, the progressive functionalization of the biosensor is analyzed from the bare metasurface to the fully modified THA configuration. The resonance wavelength displays a continuous redshift from 1378 nm to 1404 nm, accompanied by a gradual decrease in reflectance intensity (Figure 3e). These spectral trends confirm that the sequentially introduced biomolecular layers collectively modulate the optical properties of the metasurface. Notably, the streptavidin‐functionalized gold nanoparticles (SA‐AuNPs) (Δλ  ≈  3.3 nm) and TDN products (Δλ  ≈  0.9 nm) exhibit relatively minor resonance shifts, whereas the final THA nanostructures induce the most substantial overall enhancement, primarily attributed to the favorable spatial organization and electromagnetic coupling of the assembled product. Subsequently, the angle‐dependent spectral response characteristics are investigated using a single analyte system composed of AuNPs‐DNA complexes (Figure S3b). The optimal resonance angle remains at 27°, consistent with previous experimental results (Figure 3f).

Next, the influence of SA‐AuNPs size and optical density (OD) on optical response is systematically investigated. The UV absorption spectrum reveals that 20 nm SA‐AuNPs show a stable SPR peak at 525 nm, with a strong linear correlation between absorbance and concentration across the OD range of 0.1–1.0 (Figure 3g). In contrast, 40 nm SA‐AuNPs display a redshifted SPR peak at 528 nm and a slightly higher extinction coefficient due to size‐dependent plasmonic effects (Figure 3h). However, when immobilized on the metasurface, the two particle sizes show reversed performance. 20 nm SA‐AuNPs exhibit a markedly stronger increase in reflectance intensity with rising OD values, and at OD > 0.5, the Δ*λ* value significantly surpasses that of 40 nm SA‐AuNPs (*P*  <  0.0001, Figure 3i–k). This size‐dependent enhancement can be attributed to more efficient near‐field coupling effects.

### 2.3 Clinical Sample Evaluation

**FIGURE 4:**
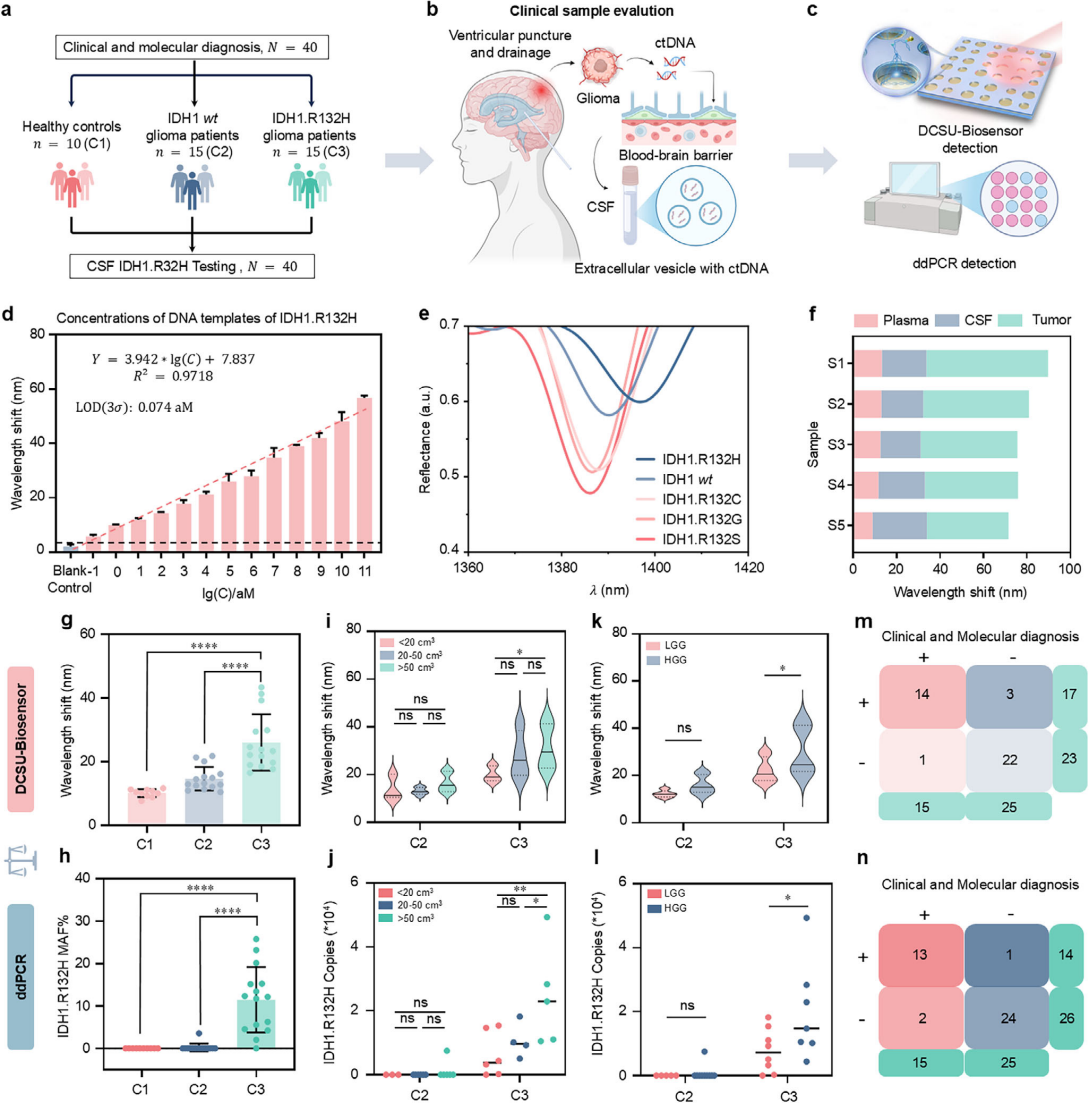
Comprehensive performance evaluation of the DCSU‐Biosensor applied to clinical samples. (a) Three parallel cohorts for clinical validation. Red: Cohort 1 (C1), healthy controls (*n*  =  10); Blue: Cohort 2 (C2), IDH1 *wt* (*n*  =  15); Green: Cohort 3 (C3), IDH1.R132H‐mutant samples (*n*  =  15). (b) Schematic illustration of tumor‐derived DNA collection from ventricular drainage CSF. Tumor cells directly release extracellular vesicles into the ventricular CSF, bypassing the blood–brain barrier. (c) Simplified schematic representation of the detection workflows for both analytical methods. (d) Correlation between the logarithmic values of IDH1.R132H gene concentration and the corresponding spectral shift signals. (e) Evaluation of the specificity of target ctDNA identification. (f) Response differences among samples from different substrates (*n*  =  5). (g–n) Performance Comparison Analysis of DCSU Biosensor and ddPCR in Clinical Sample Evaluation (g,h) IDH1.R132H mutation frequency among C1, C2, and C3. (i,j) Effect of tumor volume on measurement outcomes. (k,l) Impact of tumor stage on the results. HGG, high‐grade glioma; LGG, low‐grade glioma. (m,n) Confusion matrix comparing both methods with molecular pathology results.

The clinical performance of the DCSU‐Biosensor is evaluated using CSF specimens collected from three validation cohorts (*N*  =  40): healthy controls (Cohort 1, *n*  =  10), patients with histopathologically confirmed IDH1 *wt* gliomas (Cohort 2, *n*  =  15), and patients harboring the IDH1.R132H mutation (Cohort 3, *n*  =  15). CSF was collected from each subject for analysis. (Figure 4a). Given the restrictive nature of the blood–brain barrier, tumor‐derived cellular debris within the cranial cavity rarely enters the peripheral circulation but can circulate through the CSF. Glioma cells release a certain quantity of extracellular vesicles (EVs), which serve as key biomarkers capable of encapsulating diverse genetic material, including tumor‐derived ctDNA, thereby shielding it from enzymatic degradation [35, 36, 37]. These tumor‐derived EVs are transiently present in CSF, providing a stable source of ctDNA for molecular analysis (Figure 4b) [38]. To assess the clinical applicability and analytical accuracy of the biosensor, parallel detection is performed using both the DCSU‐Biosensor and ddPCR (Figure 4c).

Due to inherent inter‐individual variability in clinical samples, analyte concentrations exhibit significant fluctuations during detection, imposing new demands on the sensor's detection range [39, 40]. The DCSU‐Biosensor reveals a proportional increase in resonance Δλ with rising concentrations of IDH1.R132H ctDNA. Within the concentration range of 0.1 am to 100 nm, a strong linear correlation is observed between the logarithmic concentration of ctDNA and Δλ, following the regression equation:

$$\Delta \lambda =3.942*lg\left(C\right)+7.837,\;R^{2}=0.9718$$

The calculated limit of detection (LOD), determined using standard statistical analysis, is 0.074 am (Figure 4d, for detailed calculation procedures, see Note S5). This ultrahigh sensitivity, coupled with a linear dynamic range spanning 12 orders of magnitude, rivals or even surpasses state‐of‐the‐art nucleic acid testing technologies (See more details in Note S3) [23, 41, 42, 43, 44, 45, 46, 47, 48, 49, 50, 51, 52, 53], effectively encompassing the ctDNA concentration spectrum commonly observed in clinical glioma specimens.

Moreover, EVs contain cell‐free DNA derived from both normal and tumor cells, introducing compositional heterogeneity that complicates the specific capture of IDH1.R132H mutations [54, 55]. Accordingly, our investigation focuses on evaluating this variability and its influence across different sample types, forming the basis of the feasibility assessment for clinical application of the DCSU‐Biosensor. Mutations at the IDH1 R132 locus represent early oncogenic driver events in gliomagenesis [56, 57]. Distinct IDH1 mutation subtypes show markedly different biological characteristics and clinical outcomes [58]. Patients harboring the R132H mutation are more responsive to IDH1 inhibitor therapy [59], whereas those carrying R132G or R132S variants often require more aggressive chemoradiotherapy regimens [7, 8]. To evaluate the mutation‐specific recognition capability of the biosensor, reflectance spectral analyses are performed on IDH1.R132H and other IDH1 variants (*wt*, R132C, R132G, and R132S) within the wavelength range of 1360–1420 nm. The IDH1.R132H variant produces a pronounced redshift in the resonance minimum, significantly greater than that observed for other variants and the wild type (Figure 4e; Figure S4a). This distinctive optical response originates from the precise complementary design of the capture probe targeting the IDH1.R132H mutation site, which enables stable initiation of the HCR and efficient accumulation of AuNPs. By contrast, non‐target variants demonstrate mismatched hybridization, resulting in markedly attenuated optical signals.

Considering the biosensor's potential for clinical implementation, its detection performance is further evaluated across various tumor‐associated biological matrices, including plasma, CSF, and tumor tissues (Figure 4f). The ctDNA detection response in CSF and tumor tissues is significantly stronger than that observed in plasma (CSF Δλ  =  20.97  ±  2.51 nm vs. plasma Δλ  =  11.82  ±  1.76 nm). Balancing clinical feasibility and diagnostic performance, CSF is identified as the optimal detection matrix. As the direct microenvironmental fluid of the central nervous system, CSF contains higher concentrations of ctDNA with minimal interference from peripheral blood, thereby providing a more accurate molecular representation of gliomas. Although CSF sampling is mildly invasive, it remains substantially less invasive than tissue biopsy and enables real‐time molecular monitoring, including postoperative recurrence surveillance [18, 60]. Significantly, when we attempted to use this biosensor to detect DNA in tumor tissue samples, the Δλ is substantial, yet the measurement results remained within the range.

Following the extraction of circulating cell‐free DNA from bodily fluids via exosomes, quantitative PCR was employed to determine the copy number of the IDH1 transcript (Figure S4b). The results show that the mean IDH1 mRNA copy number in the CSF of glioma patients is 95567 ± 82636 copies/mL (*n*  =  30), which is significantly higher than that in healthy controls (99 ± 98 copies/mL, *n*  =  10; *P*  ≤  0.0001). CSF samples from glioma patients are collected intraoperatively from the cisterns prior to tumor manipulation, whereas control samples are obtained via lumbar puncture. This difference in sampling sites may partially explain the observed variability in IDH1 mRNA copy numbers.

Subsequently, IDH1.R132H mutation sequences are examined across all three cohorts. In Cohort 3, the biosensor detected a Δλ value of 26.04 ± 8.82 nm, while ddPCR quantified 13,539 ± 12,784 copies/mL (Figure 4g,h). These two sets of data do not exhibit a strict linear correlation, which may be attributed to the high sensitivity of the DCSU biosensor introducing so‐called “noise” in the low‐concentration range. Nonetheless, both the DCSU‐Biosensor and ddPCR consistently demonstrate statistically significant differences in IDH1.R132H mutation abundance between Cohort 3 and Cohorts 1 and 2.

To further elucidate the biosensor's capability in interpreting clinical information, subgroup analyses are performed based on clinical characteristics (see more details in Table S2), with results validated using both the biosensor and ddPCR. Overall, both approaches display consistent trends, with higher IDH1.R132H copy numbers observed in (i) tumors >50 cm^3^ and (ii) high‐grade gliomas (HGG) (Figure 4i–l). This result indicates a positive correlation between IDH1 mutation abundance and both tumor size and malignancy grade, consistent with previous clinical studies. It is noteworthy that differences were observed between biosensor results and ddPCR findings across different age groups, while no such differences were found between gender groups (Figure S4c–f).

This suggests the potential presence of age‐related matrix effects in CSF samples, with elevated protease activity in the CSF of elderly patients possibly contributing to this phenomenon. Furthermore, its signal intensity reflects mutation abundance in correlation with tumor malignancy and size, providing valuable biological insights for clinical prognostic assessment. In addition, DCSU‐Biosensor maintains strong clinical relevance across preoperative, postoperative, and follow‐up stages. This demonstrates its significant potential for monitoring tumor recurrence and detecting residual disease after surgery (Figure S5a,b).

The DCSU‐Biosensor and ddPCR exhibit slight deviations in absolute quantification, but both methods demonstrate comparable discriminatory power in distinguishing healthy individuals from IDH1.R132H mutant and IDH1 *wt* glioma patients. This consistency supports their utility in accurate clinical cohort stratification and reliable molecular subtyping of gliomas. Based on confusion matrix analysis comparing both methods, performance metrics revealed that the biosensor possesses several advantages over ddPCR, including higher sensitivity, the absence of false negatives, a shorter detection time (1–1.5 h) [61, 62], and label‐free detection

(Figure 4m,n). Although its specificity was slightly lower than that of ddPCR, its 93.33% sensitivity minimizes the risk of missed diagnoses (Table S3).

## 3 Conclusion

In summary, the proposed label‐free dual‐channel co‐sensing strategy achieves a major breakthrough in the field of biosensing for molecular diagnostics. By leveraging the dissipative field‐enhanced optical platform mechanism based on structural and momentum‐cooperative symmetry breaking, combined with biomolecular amplification effects realized through DNA nanostructure self‐assembly, this strategy achieves an exceptional balance of sensitivity, specificity, and practicality. Requiring only minimal sample volumes, it simultaneously achieves an ultra‐low detection threshold and an exceptionally broad linear dynamic range. This enables reliable identification of glioma subtypes and provides valuable clinical information. This strategy not only outperforms existing nucleic acid detection methods but also resolves critical discrepancies between peripheral and central tumor detection. The DCSU‐Biosensor holds significant translational potential for early glioma screening, postoperative monitoring, and broader precision oncology applications.

## 4 Experimental Section/Methods

### 4.1 Simulation Methods

The numerical simulations were conducted using the finite integration technique (FIT) in the frequency domain. Floquet periodic boundary conditions were applied along both the x‐ and y‐axes, while the z‐axis boundaries are defined as open (space) and open, respectively. The incident electromagnetic wave was directed along the negative z‐axis, with variable incident angles applied to excite resonant modes within the metasurface unit cell (meta‐atom). The refractive index of silicon nitride (Si_3_N_4_) was experimentally determined via spectroscopic ellipsometry (see details in Note S5), whereas the refractive index of silicon dioxide (SiO_2_) was set to 1.45. The optical constants of gold (Au) were adopted from the classical dataset reported by Johnson and Christy (1972) [63].]

### 4.2 Device Fabrication

The metasurface arrays were fabricated using electron beam lithography (EBL) and reactive ion etching (RIE), yielding a final array area of 100 µm × 100 µm. Initially, a 100 nm‐thick gold film was deposited onto 1 cm × 1 cm double‐sided, polished fused silica substrates via magnetron sputtering [64]. Subsequently, a 600 nm‐thick Si_3_N_4_ layer was deposited on the gold surface using plasma‐enhanced chemical vapor deposition. The designed nanostructure pattern was then defined using EBL exposure and development, followed by RIE etching to form the quadrumer hole‐type metasurface.

### 4.3 THA Complex Assembly on the Metasurface

All oligonucleotides, including TDN component strands, thiol‐modified strands, capture probes, and hairpins (H1/H2), were synthesized and high‐performance liquid chromatography–purified by Tsingke Biotechnology Co., Ltd. (Beijing, China). Detailed oligonucleotide sequences are available in Table S4. AuNPs with nominal diameters of 20 and 40 nm were obtained from Sigma–Aldrich (St. Louis, MO, USA). All buffers and solutions were prepared using nuclease‐free water (Solarbio, Beijing, China) and analytical‐grade reagents.

To prepare SA–AuNPs, 10 µL of SA (1 mg mL^−^^1^ in 10 mm sodium phosphate buffer, pH 7.4) was added to 500 µL of AuNPs solution. The mixture was incubated on ice for 1 h to facilitate physical adsorption of SA molecules onto the AuNPs surface. The final concentration of SA in the reaction mixture was 3.6 × 10^−^^7^ m. Unbound SA was removed by centrifugation at 12 500 rpm for 30 min at 23°C, and the resulting pellet of SA‐AuNP conjugates was redispersed in 90 µL of nuclease‐free water.

Metasurface chips featuring patterned gold regions were sequentially ultrasonicated in acetone and ethanol (10 min each), rinsed thoroughly with deionized water, and subjected to oxygen plasma treatment for 5 min to activate the surface and remove organic contaminants. Thiol‐functionalized TDNs were self‐assembled from equimolar mixtures of four component strands (final concentration 1 µm each) in TM buffer (20 mm Tris‐HCl, 50 mm MgCl_2_, pH 8.0) via an annealing process consisting of heating to 95°C for 10 min followed by gradual cooling to 4°C at −1°C/min. Cleaned and activated metasurface chips were incubated with 0.5 µm thiolated TDN solution at 37°C for 2 h to enable thiol‐gold coordination anchoring at the three basal vertices of each TDN. After incubation, unbound TDNs were removed by washing with PBS containing 0.05% Tween‐20 (PBST). To evaluate the stability and repeatability of the metasurface under cleaning treatment and prolonged measurement time, we characterized the reflectance spectra after cleaning and one month later. As shown in Figure S7, the metasurface maintains structural integrity and optical stability during routine cleaning procedures and time‐dependent measurements.

To minimize nonspecific adsorption, the surface was passivated with 1 mm 6‐mercapto‐1‐hexanol for 30 min at room temperature. Subsequently, hairpins (H1/H2, each 1 µm) were added to the surface. For target detection, a synthetic IDH1.R132H oligonucleotide (target mimic) was introduced onto the TDN‐modified metasurface and incubated at 37°C for 1 h, initiating on‐surface HCR and the formation of extended double‐stranded DNA polymers. The SA‐AuNPs were then introduced onto the metasurface and incubated for 30 min at room temperature. Following the step, the chips were stringently washed with PBST to remove non‐specifically bound molecules, gently dried under nitrogen, and immediately subjected to optical characterization and spectral measurement.

### 4.4 Ethical Approval

This study was conducted in accordance with the ethical standards of the Institutional Review Board and Ethics Committee of Peking University Third Hospital (Beijing, China) and was approved under approval nos. M20250800 and 20250903‐02135‐0251. All research procedures complied with institutional and national ethical guidelines. Written informed consent was obtained from all participants prior to study enrollment. Clinical Study Registration: Not applicable. This study was not a prospective interventional clinical trial and therefore did not require registration.

### 4.5 Patient Cohort and Sample Collection

A retrospective cohort comprising 30 glioma patients admitted to the Department of Neurosurgery at Peking University Third Hospital between September 2018 and September 2024 was included in this study. The median age at diagnosis was 12.0 years (range, 3.7–56.0 years). All patients underwent standardized preoperative magnetic resonance imaging (MRI) for clinical evaluation.

### 4.6 CSF Sampling

Intraoperative CSF samples (2‐3 mL) were collected from 30 patients during tumor resection or ventriculoperitoneal shunt placement, prior to direct tumor manipulation to avoid contamination. Control CSF samples were obtained via lumbar puncture from age‐matched healthy volunteers (*n*  =  10). All samples were processed under identical laboratory conditions to minimize preanalytical variability.

### 4.7 Tumor Tissue and Peripheral Blood Sampling

Fresh tumor tissues were acquired via surgical resection or open biopsy. All specimens were reviewed by board‐certified neuropathologists, and hematoxylin and eosin (H&E) staining confirmed that viable tumor cells accounted for more than 70% of each sample. Matched peripheral blood samples were collected from all patients for comparative analyses.

### 4.8 MRI Acquisition and Tumor Volume Quantification

All participants underwent standardized preoperative MRI scans using 3.0‐T systems. Tumor volumes were quantified through manual segmentation using 3D Slicer software (version 4.8.0). Delineation of tumor margins was based on contrast‐enhanced T1‐weighted imaging (T1WI) and T2‐weighted or T2‐fluid‐attenuated inversion recovery (T2WI/T2‐FLAIR) sequences. For lesions with heterogeneous enhancement, tumor boundaries were defined by hyperintense regions on T2WI/T2‐FLAIR. Image segmentation was performed independently by an experienced neurosurgeon and validated by a senior author to ensure reproducibility and inter‐rater reliability.

### 4.9 Isolation of ctDNA

Freshly collected CSF samples were maintained at 4°C until centrifugation at 1 400 rpm for 5 min. Supernatants were transferred to sterile cryotubes, aliquoted (3.5 mL per tube), and stored at −80°C until analysis. Prior to ctDNA extraction, samples were thawed in a 37°C water bath. ctDNA was isolated using the QIAGEN QIAsymphony SP automated platform and the QIAsymphony DSP Circulating DNA Kit (Cat. No. 937556).

Whole blood samples were collected into Streck Cell‐Free DNA BCT tubes and processed within 2 h. Plasma was separated via two‐step centrifugation: first at 800 × g for 10 min to remove blood cells, followed by a second spin at 18 000 × g for 10 min to eliminate residual cellular debris. Cell‐free plasma (3.5 mL) was stored at −80°C and processed using the same ctDNA extraction protocol.

For tumor tissue samples, genomic DNA was extracted from formalin‐fixed, paraffin‐embedded specimens. Deparaffinization was performed using mineral oil, followed by enzymatic digestion with Proteinase K to degrade proteins and release nucleic acids. DNA purification and extraction were conducted using the QIAsymphony SP system according to the manufacturer's instructions.

### 4.10 DCSU‐Biosensor Detection

The processed clinical samples were pipetted (0.5–2.5 µL) onto the platform surface for incubation. Following the formation of the DNA self‐assembled complex on the surface, impurities were removed by cyclic washing with acetone, anhydrous ethanol, and deionized (DI) water. Subsequently, the platform was placed on a reflective variable‐angle near‐infrared measurement stage for spectral detection (see Figure S8).

### 4.11 ddPCR Detection

ddPCR assays were performed to quantify the IDH1.R132H mutation. Each 20 µL reaction contained ddPCR Supermix, IDH1.R132H‐specific primers and probes, template DNA, and nuclease‐free water. Following vortex mixing and brief centrifugation, the reaction mixtures were loaded into a DG32 droplet generation cartridge, and 70 µL of droplet generation oil was added to each corresponding well. The cartridge was sealed with a droplet generation cap and placed into the Bio‐Rad QX600 Droplet Generator to form uniform emulsified droplets.

Following droplet generation, approximately 40 µL of emulsion was carefully transferred to a 96‐well PCR plate, sealed using a PX1 PCR Plate Sealer at 180°C for 4–5 s, and subjected to PCR amplification according to manufacturer‐recommended cycling conditions. Fluorescence detection and data acquisition were subsequently conducted using the QX600 Droplet Reader (Bio‐Rad), and results were analyzed with QuantaSoft Analysis Pro software.

## Conflicts of Interest

The authors declare no conflicts of interest.

## Supporting information




**Supporting File**: advs75443‐sup‐0001‐SuppMat.docx.

## Data Availability

The data that support the findings of this study are available from the corresponding author upon reasonable request.
