# Supplementary material for: A Dual‐Channel Synergistic Ultrasensitive Biosensor for Tumor Liquid Biopsy
Source: Adv Sci (Weinh). 2026 Apr 27;13(41):e75443. doi: 10.1002/advs.75443 (PMC13335429; doi:10.1002/advs.75443)
Supplement: Supplementary file 1 — Supporting File: advs75443‐sup‐0001‐SuppMat.docx. [file ADVS-13-e75443-s001.docx]

**Supporting Information**

**A Dual-Channel Synergistic Ultrasensitive Biosensor for Tumor Liquid Biopsy**

Yu Sun^1,4,#^, Jing Zhang^2,6,#^, Chenqing Liu^3,#^, Jing Lou^2*^, Sihui Wang^1,5^, Xiangtian Ji^1,5^, Xiaofang Zhao^1,5^, Shirui Xu^4^, Jingyu Feng^1,5^, Bin Liu^1,5^, Chao Chang^2,7,*^, Qinggang Ge^4,*^, Jun Yang^1,5*^

^1^Department of Neurosurgery, Peking University Third Hospital, Beijing 100089, P.R. China

^2^Innovation Laboratory of Terahertz Biophysics, National Innovation Institute of Defense Technology, Beijing 100071, P. R. China

^3^Department of Otorhinolaryngology, Head and Neck Surgery, The Sixth Medical Center of the Chinese PLA General Hospital, Beijing 100853, China

^4^Department of Intensive Care Unit, Peking University Third Hospital, Beijing 100089, P.R. China

^5^Center for Precision Neurosurgery and Oncology of Peking University Health Science Center, Beijing 100089, P.R. China

^6^Air and Missile Defense College, Air Force Engineering University, Xi’an 710051, P. R. China.

^7^School of Physics, Peking University, Beijing 100081, P. R. China

*Correspondence and requests for materials should be addressed to J.L. (email: [loujing9486@163.com](mailto:loujing9486@163.com)) or to C.C. (email: [gwyzlzssb@pku.edu.cn](mailto:gwyzlzssb@pku.edu.cn)) or to QG.G. ([qingganggelin@126.com](mailto:qingganggelin@126.com)) or to J.Y. ([yangjbysy@bjmu.edu.cn](mailto:yangjbysy@bjmu.edu.cn)).

^#^ These authors have equal contribution for the manuscript.

Supplementary Note 1 Explaining the resonant modes

Supplementary Note 2 The preparation process of DNA self-assembled nanostructures

Supplementary Note 3 Comparison with emerging nucleic acid testing technologies

Supplementary Note 4 Clinical sample information collection and comparative study

Supplementary Note 5 Methods

**Supplementary Note 1. Explaining the resonant modes**

To further analyze the resonant modes of the dual-channel, synergistic, symmetry-broken metasurface, we perform a multipole decomposition to evaluate the contributions of different multipole modes to the resonance. The calculation formula is as follows (Cartesian coordinate system, α, β = x, y, z):

$$\boldsymbol{P}=\frac{1}{i\omega}\int\boldsymbol{J}d\boldsymbol{r}$$

$$\boldsymbol{T}=\frac{1}{10c}\int\left[ \left( \boldsymbol{r\cdot J} \right)r-2\boldsymbol{r}^{2}\boldsymbol{J} \right]d\boldsymbol{r}$$

$$\boldsymbol{M}=\frac{1}{2c}\int\left( \boldsymbol{r}\times\boldsymbol{J} \right)d\boldsymbol{r}$$

$$\boldsymbol{Q}_{\alpha,\beta}^{\left( e \right)}=\frac{1}{2i\omega}\int\left[ r_{\alpha}J_{\beta}+r_{\beta}J_{\alpha}-\frac{2}{3}\delta_{\alpha,\beta}\left( \boldsymbol{r\cdot J} \right) \right]d\boldsymbol{r}$$

$$\boldsymbol{Q}_{\alpha,\beta}^{\left( m \right)}=\frac{1}{3c}\int\left[ \left( \boldsymbol{r}\times\boldsymbol{J} \right)_{\alpha}r_{\beta}+\left( \boldsymbol{r}\times\boldsymbol{J} \right)_{\beta}r_{\alpha} \right]d\boldsymbol{r}$$

$$I_{P}=\frac{2\omega^{4}}{3c^{3}}\left| \boldsymbol{P} \right|^{2}$$

$$I_{T}=\frac{2\omega^{6}}{3c^{3}}\left| \boldsymbol{T} \right|^{2}$$

$$I_{M}=\frac{2\omega^{4}}{3c^{3}}\left| \boldsymbol{M} \right|^{2}$$

$$I_{Q^{\left( e \right)}}=\frac{\omega^{6}}{5c^{5}}\left| \boldsymbol{Q}_{\alpha,\beta}^{\left( e \right)} \right|^{2}$$

$$I_{Q^{\left( m \right)}}=\frac{\omega^{6}}{20c^{5}}\left| \boldsymbol{Q}_{\alpha,\beta}^{\left( m \right)} \right|^{2}$$

where ***r*** and ***J*** represent the position vector and the current density, and the main types of multipole moments, including ***P*** (electric dipole moment), ***T*** (toroidal dipole moment), ***M*** (magnetic dipole moment), $\boldsymbol{Q}_{\alpha,\beta}^{\left( e \right)}$ (electric quadrupole moment), and $\boldsymbol{Q}_{\alpha,\beta}^{\left( m \right)}$ (magnetic quadrupole moment) are considered. The multipole scattering intensities corresponding to *δ* = 100 nm under different incident angles are calculated, as shown in **Fig. S1**. The resonance is primarily attributed to the higher-order electric quadrupole mode. As the incident angle increases, the electric quadrupole intensity first decreases and then increases, reaching its maximum at *θ* = 27°, which exhibits the same trend as the overall resonance strength.

**
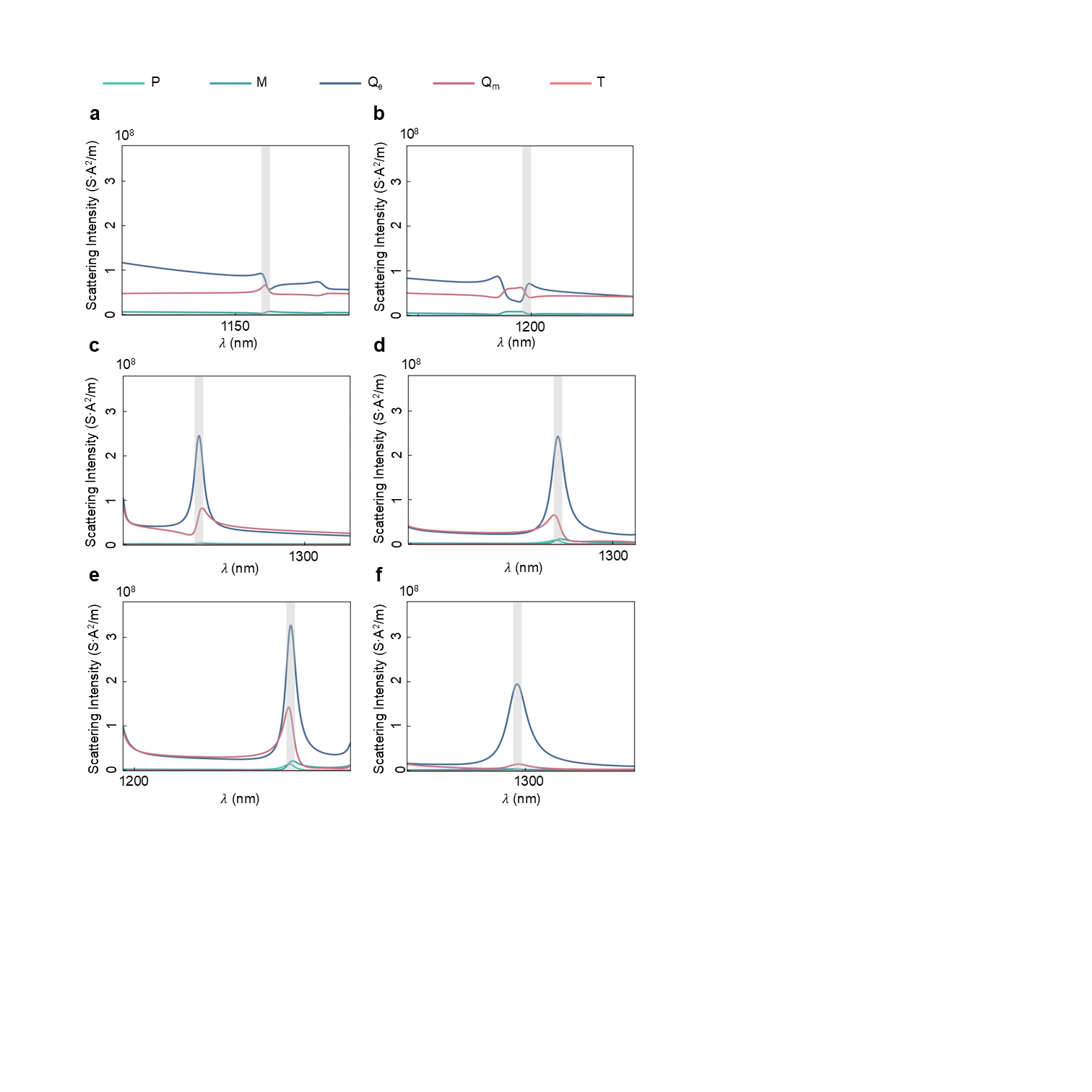
Figure S1** Multipole scattering intensity maps: **a** *δ* = 100 nm, *θ* = 0°; **b** *δ* = 100 nm, *θ* = 5°; **c** *δ* = 100 nm, *θ* = 15°; **d** *δ* = 100 nm, *θ* = 25°; **e** *δ* = 100 nm, *θ* = 27°; **f** *δ* = 100 nm, *θ* = 35°.

**Supplementary Note 2. The preparation process of DNA self-assembled nanostructures**


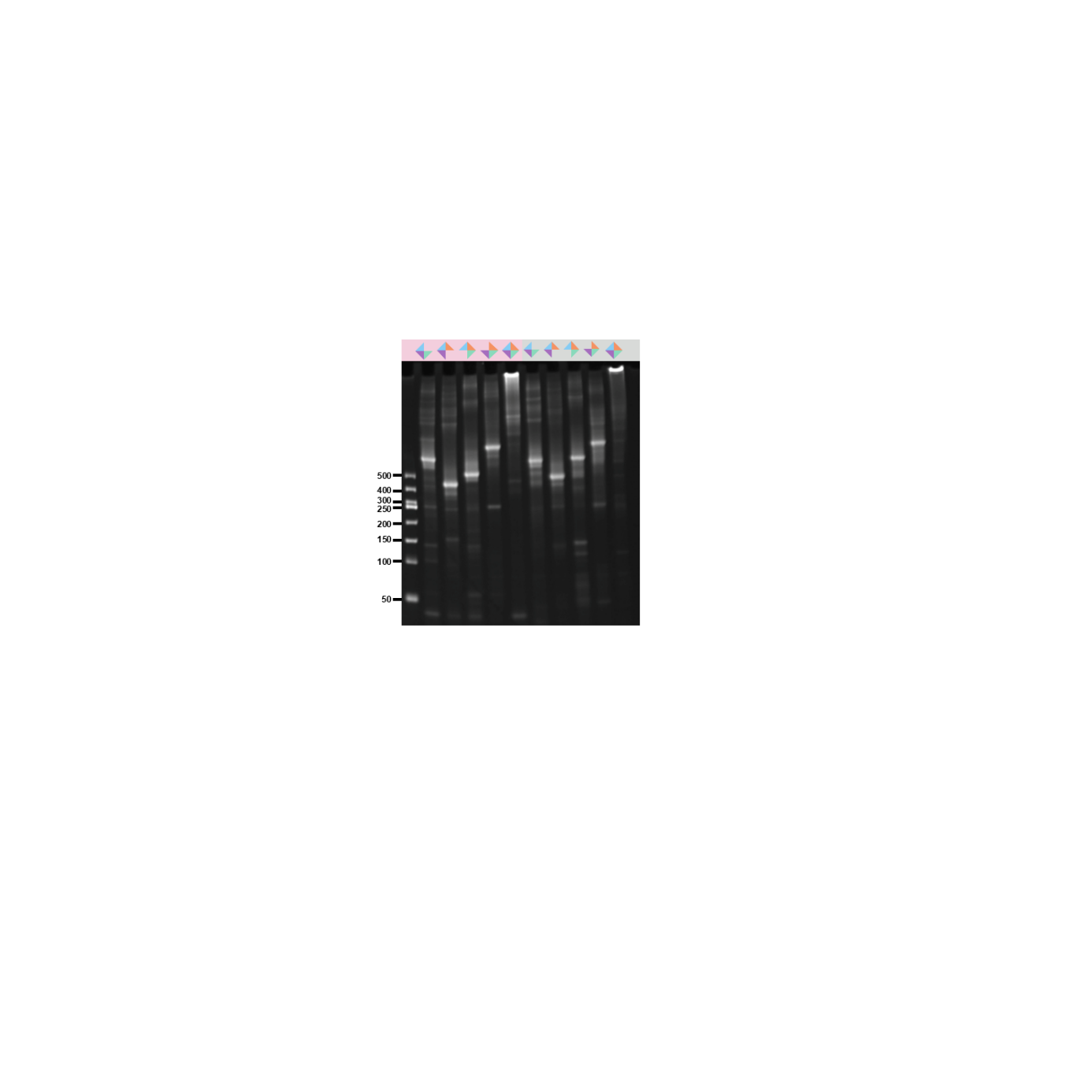
To obtain sequences better suited for constructing tetrahedral DNA (TDN) structures, we investigate the effect of CG content on TDN synthesis efficiency. Polyacrylamide gel electrophoresis (PAGE) analysis shows that TDN structures composed of strands 5–8, which have higher GC content, exhibit greater structural stability than those formed by strands 1–4. We further present electron micrographs of the self-assembled nanostructures of TDN. In Fig. S3a, the black arrows in the figure denote SA-AuNPs, while the red arrows indicate TDN complexes. Both are SA-biotin conjugates, with the complex surfaces capped with 6-hydroxy-1-hexanethiol. Fig.S3b indicates that the absorption spectrum of the DNA-AuNPs complex decreases at 520 nm.

**Figure S2** TDN composite structure PAGE analysis diagram.

**
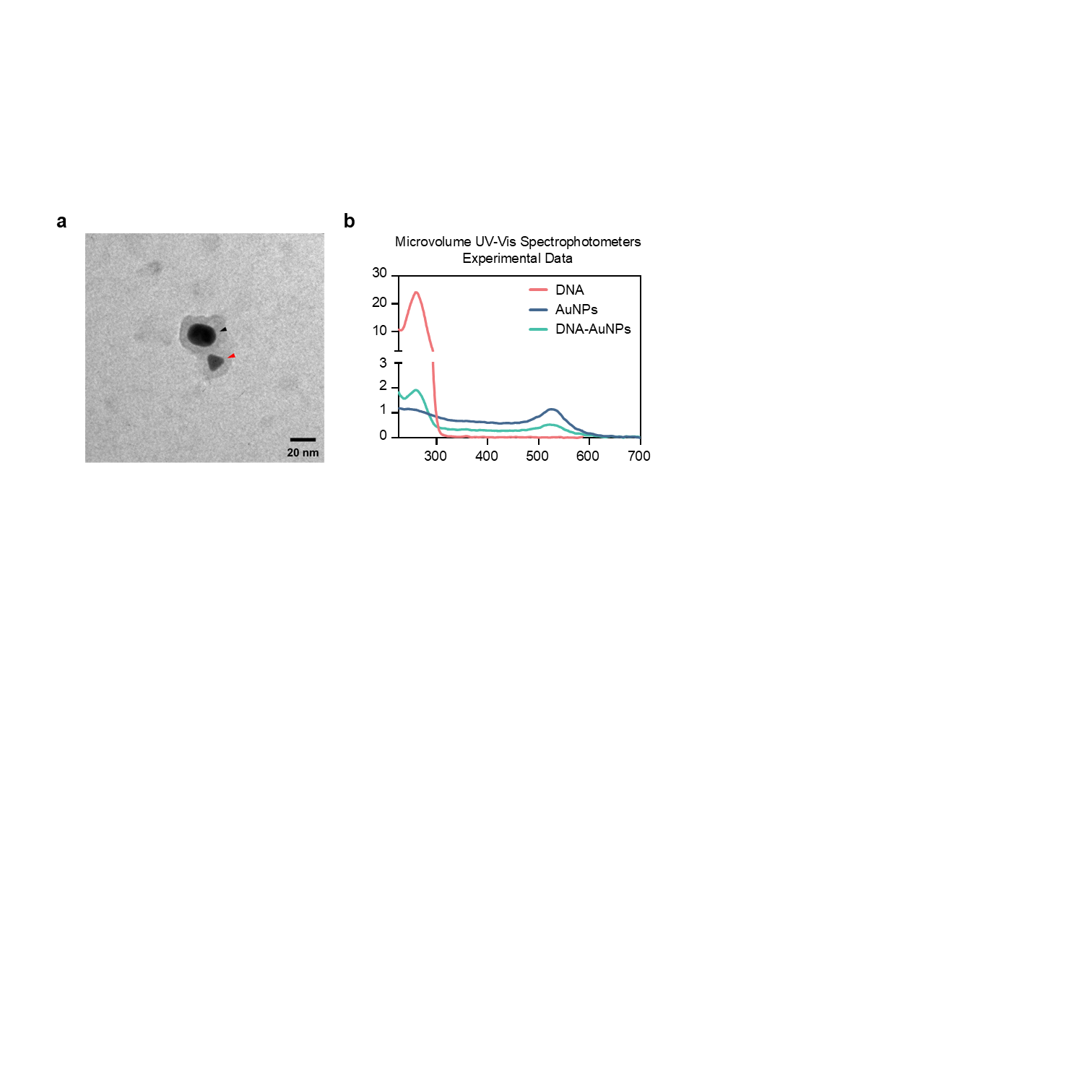
**

**Figure S3** **a** Transmission electron microscopy (TEM) observation of the TDN complex. **b** UV-visible molecular absorption spectra of DNA, AuNPs, and DNA-AuNPs.

**Supplementary Note 3.** **Comparison with emerging nucleic acid testing technologies**

We have compiled a list of highly cited and high-impact-factor papers recently published on nucleic acid detection. The results indicate that the labelling-free dual-linkage cooperative sensing strategy outperforms other approaches in terms of limit of detection (LOD), linear response range, and sample requirements. Furthermore, this emerging nucleic acid detection technology has been applied to central nervous system-related diseases.

**Table S1** The list for the performance of various emerging nucleic acid biosensors

| **No.** | **Ref** | **Target** | **Primary**  **lesion**  **(Central (C.)/Peripheral (P.))** | **Signal amplification strategy** | **LOD ^a)^** | **Linear range**  **(X order of magnitude)** | **Clinical**  **sample**  **require**  **ment** |
| --- | --- | --- | --- | --- | --- | --- | --- |
| 1 | Nat.commun. (2020), 11, 1543 | miRNA/DNA | P. | graphene FET ^b)^ | 0.6/  20 aM | 10^-18^-10^-6^ (12) | 50 μL |
| 2 | Adv.Funct.Mater. (2022), 32, 2109728 | miR-21 | P. | TDN-HCR | 0.1 nM | 10^-10^-10^-8^(2) | 50 μL |
| 3 | Sci.Adv. (2022), 8, eabn2378 | SARS-CoV-2  (S-RBD) | P. | mini-TIRF microscope ^c)^ | 30 fM | 10^-18^-10^-10^(10) | 10 μL |
| 4 | Nat.commun. (2023), 14, 4486 | SARS-CoV-2  E and ORF1b | P. | QBIC | 8 fM | 10^-15^-10-^6^ (9) | 1 mL |
| 5 | Nat.commun. (2023), 14, 7504 | Bladder cancer  (miR-19a) | P. | CRISPR technology | 856 aM | 10^-15^-10^-11^ (4) | 2 μL |
| 6 | Adv.Mater. (2023), 35, 2304119 | breast cancer  ctDNA/  an estrogen receptor ERβ/monkeypox virus antigen A35R | P. | TDN&  Electrochemistry | 0.21/  6.74/  991 aM | 10^-14^-10^-10^(4) | 80 μL |
| 7 | Biosens.  Bioelectron. (2023), 237, 115557 | miR-21 | P. | Graphdiyne &DNA nanoring | 35.1/  61.6 aM | 10^-16^-10^-10^(6)/  10^-16^-10^-8^(8) | ND ^d)^ |
| 8 | Nat.commun. (2024), 15, 1818 | ctDNA (SNP)/H.Pylori DNA and SARS-CoV-2 RNA | P. | CRISPR technology | 1 aM/  100 aM | 10^-18^-10^-9^ (9) | 10 μL |
| 9 | Nat.commun. (2024), 15, 1936 | EBV cfDNA | P. | TDN | 0.3 fM | 3×10^-16^-3×10^-13^ (3) | 10 μL |
| 10 | Laser & Photonics Rev. (2024), 18, 2400035 | T790M EGFR/  G12C KRAS | P. | TDN and CRISPR technology | 0.35/  0.14 aM | 10^-20^-10^-10^ (10) | ND |
| 11 | Sci.Adv. (2025), 11, eadu2271 | ASFV DNA and SARS- CoV- 2 RNA fragments | P. | CRISPR technology | 0.25 aM | 2.5×10^-19^-1×10^-13^ (6) | 11 μL |
| 12 | Adv.Mater. (2025), 37, 2501378 | miR-21 | P. | DNAzyme | 1.26 pM | 10^-9^-10^-7^ (2) | 10 μL |
| 13 | ACS.nano. (2025), 19,  5526 | EGFR L858R and  T790M | P. | Charged liposomes and Microfluid | 1 fM | 10^-15^-2×10^-6^ (9) | 20 μL |
| 14 | Adv.Funct.Mater. (2025), 36, e12115 | miR-21 | P. | supramolecular  hydrogel | 25 nM | 2.5×10^-8^-1.25×10^-7^(1) | 100 μL |
| 15 | Adv.Sci. (2025), early view, e19758 | miR-125b-5p, miR-21-5p and miR-155-5p | P. | anionic liposome | 16 aM, 50.3 fM and 72.2 fM | 1.6×10^-17^-10^-8^(9) | 20 μL |
| **16** | **This work** | **Glioma Mutation Genes IDH1.R132H** | **C.** | **THA&QBIC** | **74 zM** | **10^-19^-10^-7^ (12)** | **<1 ul** |

^a)^ nM (10^-9^ M)>pM (10^-12^ M)>fM (10^-15^ M)>aM (10^-18^ M)>zM (10^-21^ M), with a difference of 10^3^; ^b)^ Field Effect Transistor; ^c)^ Miniature total internal reflection fluorescence microscopy; ^d)^ Not mentioned;

**Supplementary Note 4.** **Clinical sample information collection and comparative study**

We have collected key information from 30 clinical samples and grouped them by age and gender as detailed in Table S2. Findings reveal no significant differences in most age and gender cohorts in Fig.S4c-f, consistent with anticipated outcomes. However, ddPCR analysis demonstrates significant variation within the IDH1 mutation group, potentially attributable to older patients typically presenting with more aggressive tumors. To further evaluate the clinical applicability of the DCSU-Biosensor, we analyzed glioma patient samples collected at different clinical stages in Fig.S5a, b, including pre-operative, post-operative, and follow-up conditions (*n* = 5). The resonance wavelength shift (Δ*λ*) exhibited a decreasing trend after surgery, consistent with reduced tumor-associated ctDNA burden. These results support the potential of the DCSU-Biosensor platform for micro-volume dynamic monitoring of treatment response and recurrence risk.

| **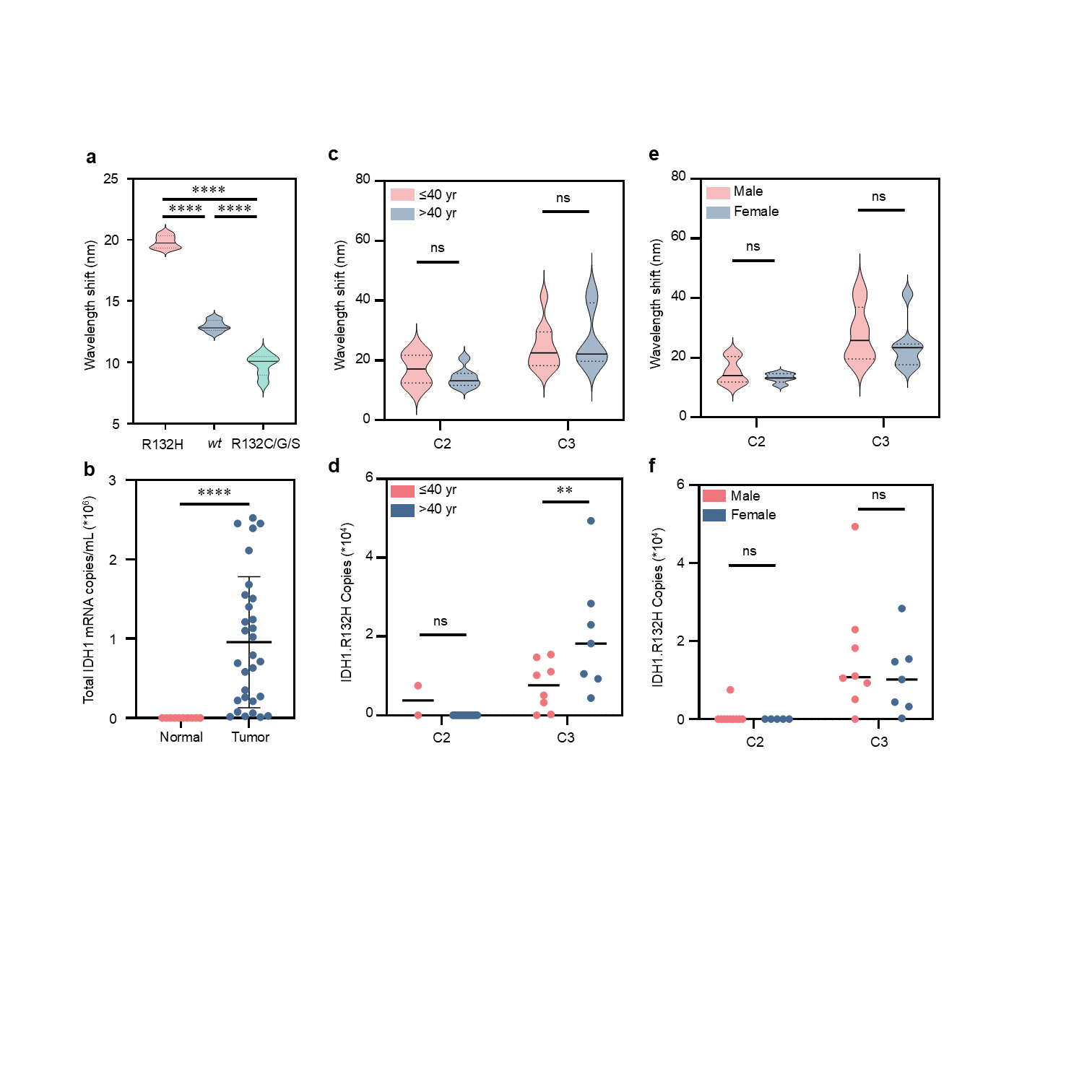** |
| --- |

**Figure S4** **a** Specificity analysis of DCSU-Biosensor detection for IDH1.R132H mutations ($n = 5$). **b** Differences in expression of the IDH1.R132H mutant gene between normal and tumor CSF. **c-f** Impact of age and gender on the results obtained from both detection methods. **(c, d)** DCSU-Biosensor. **(e, f)** ddPCR.

| 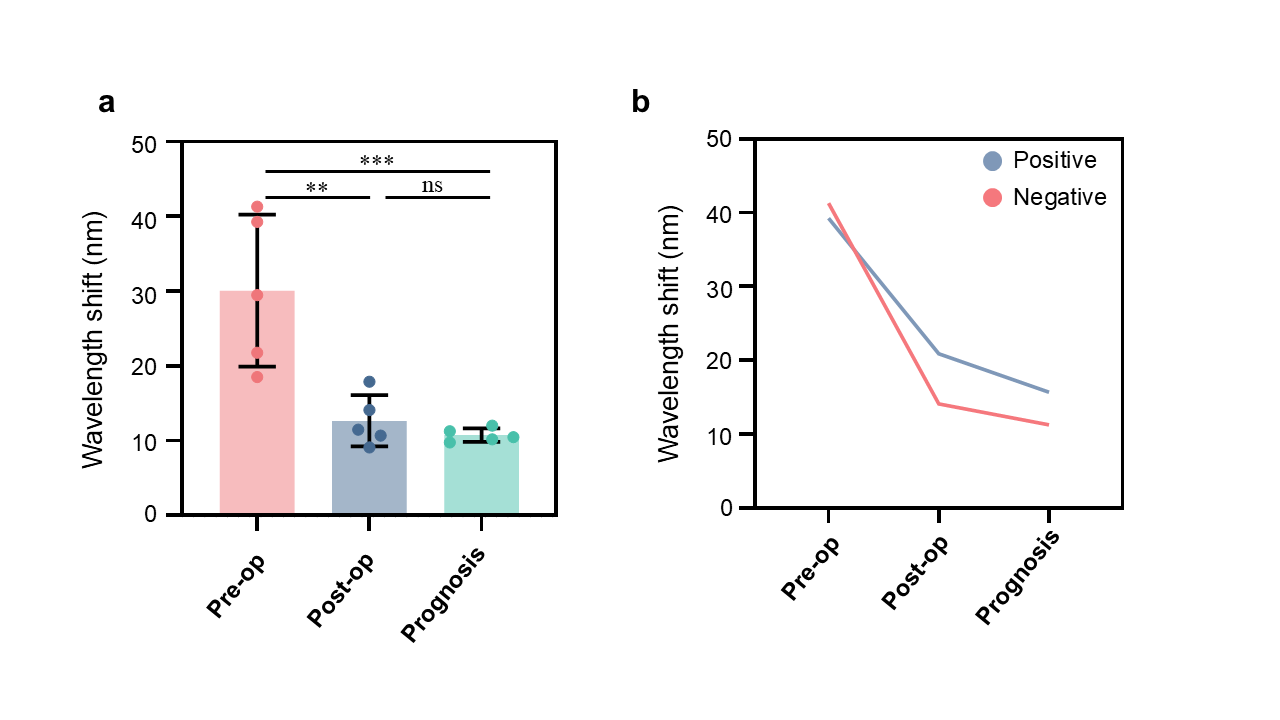 |
| --- |

**Figure S5** Dynamic monitoring of glioma ctDNA burden across perioperative and follow-up stages using the DCSU-Biosensor. **a** Δ*λ* values of glioma patients across pre-operative, post-operative, and follow-up stages, showing an overall decrease after surgery. **b** Trend analysis of Δ*λ* across clinical stages, illustrating its potential for dynamic monitoring of tumor burden and recurrence risk.

**Table S2** IDH1.R132H mutant study population demographics and disease parameters

| **Parameter** | **IDH1 wild-type (N = 15)** | **IDH1.R132H (N = 15)** |
| --- | --- | --- |
| **Age (yr)** |  |  |
| ≤ 40 | 2 (13.3) | 7 (46.7) |
| ≥ 40 | 13 (86.7) | 8 (53.3) |
| **Gender** |  |  |
| Male | 10 (66.7) | 8 (53.3) |
| Female | 5 (33.3) | 7 (46.7) |
| **WHO Grade^a)^** |  |  |
| Low-Grade | 5 (33.3) | 8 (53.3) |
| High-Grade | 10 (66.7) | 7 (46.7) |
| **Diagnosis** |  |  |
| Astrocytoma, Grade 2-3 | 2 (13.3) | 6 (40.0) |
| Oligodendroglioma,  Grade 2-3 | 0 (0.0) | 5 (33.3) |
| GBM, Grade 4 | 7 (46.7) | 2 (13.3) |
| Others | 6 (40.0) | 2 (13.3) |
| **Cortical Location** |  |  |
| Frontal | 4 (26.7) | 7 (46.7) |
| Parietal | 4 (26.7) | 1 (6.6) |
| Temporal | 1 (6.6) | 3 (20.0) |
| Others | 6 (40.0) | 4 (26.7) |
| **Tumor Volume (cm^3^)** |  |  |
| < 20 | 3 (20.0) | 6 (40.0) |
| 20-50 | 6 (40.0) | 4 (26.7) |
| > 50 | 6 (40.0) | 5 (33.3) |
| **Recurrence** |  |  |
| Yes | 1 (6.6) | 2 (13.3) |
| No | 14 (99.3) | 13 (86.7) |
| **MGMT status ^b)^** |  |  |
| methylated | 7 (46.7) | 8 (53.3) |
| unmethylated | 8 (53.3) | 7 (46.7) |
| **TP53 status ^c)^** |  |  |
| wild-type | 7 (46.7) | 6 (40.0) |
| mutant | 8 (53.3) | 9 (60.0) |
| **ATRX status ^d)^** |  |  |
| wild-type | 7 (46.7) | 7 (46.7) |
| mutant | 8 (53.3) | 8 (53.3) |
| **Ki-67 ^e)^** |  |  |
| < 5% | 2 (13.3) | 3 (20.0) |
| 5-20% | 5 (33.3) | 7 (46.7) |
| > 20% | 8 (53.3) | 5 (33.3) |

^a)^ WHO World Health Organization; ^b)^ MGMT O6-methylguanine-DNA methyl-transferase; ^c)^ TP53 Tumor Protein 53; ^d)^ ATRX α-thalassemia/mental-retardation-syndrome-X-linked gene; ^e)^ Ki-67 Proliferation Kiel-67.

**Table S3** Performance Comparison and Clinical Relevance of DCSU-Biosensor vs. ddPCR for IDH1.R132H Mutation Detection

| **Metric** | **DCSU-Biosensor** | **ddPCR** | **Clinical Relevance** |
| --- | --- | --- | --- |
| Sensitivity | **93.33%** | **86.67%** | The high sensitivity of DCSU-Biosensor ensures a low false-negative rate, which is critical for early detection of the IDH1 R132H mutation. |
| Specificity | 88.00% | 96.00% | The DCSU-Biosensor exhibits marginally lower specificity compared to ddPCR; nevertheless, it retains significant utility in clinical settings as a supplementary diagnostic tool. |
| Positive Likelihood Ratio | 7.78 | 21.67 | ddPCR demonstrates superior applicability for definitive clinical diagnosis. |
| Negative Likelihood Ratio | 0.08 | 0.14 | The DCSU-Biosensor demonstrates superior applicability for screening and early detection purposes. |
| Youden's Index | 0.81 | 0.83 | Both methods show excellent overall performance. |
| Test duration | **1-1.5 h** | **2-4 h** | Under identical sample pretreatment conditions, the DCSU-Biosensor demonstrates significantly shorter amplification time while operating in a label-free manner, thereby mitigating potential signal interference inherent to conventional detection methodologies. |

**Supplementary Note 5.** **Methods**

*Simulation*

To ensure the accuracy of the simulations, we measured the refractive index parameters of silicon nitride before pattern etching using an ellipsometer, as shown in Fig. S6. Fig. S6a presents the measured real part of the refractive index, while Fig. S6b shows the corresponding imaginary part. In the finite element integral method, the material parameters corresponding to the relevant wavelength range were employed.

*The detailed DNA sequences*

In Table S4, the probe hybridizes with the bold regions of ctDNA (IDH1.R132H). The bold regions of hairpins H1 and H2 self-hybridize to form stable hairpin structures. The underlined italicized region of ctDNA (IDH1.R132H) hybridizes with the corresponding underlined italicized region of hairpin H1, initiating the HCR. Strands 1–4 and strands 5–8 represent the four oligonucleotides used for synthesizing the DNA tetrahedron, where bold regions denote exposed probe sequences.

*DCSU-Biosensor signal stability*

To further verify device robustness, we systematically examined the influence of cleaning procedures and measurement duration on the optical response of the metasurface. The comparison spectra are shown in Fig. S7. After cleaning with piranha solution, the resonance wavelength exhibits minimal variation, indicating that the structural geometry and surface morphology are well preserved. In addition, time-dependent measurements reveal no obvious drift in the resonance position, confirming the intrinsic stability of the Q-BIC self-assembled mode under ambient conditions. The slight intensity variations observed are attributed to minor experimental fluctuations rather than structural changes.

*Measurement optical path diagram*

In this study, we constructed a reflection-type variable-angle near-infrared measurement platform (Figure S8), which consists of light source with an output wavelength range of 360–2600 nm, two wire-grid polarizers operating in the 300–3200 nm range, optical fibers, and a spectrometer capable of recording spectra from 900 to 1700 nm. During the measurement process, the vertical incidence test was employed with beam splitter. And the oblique incidence tests were performed by synchronously rotating the detector arm. To ensure measurement accuracy, each angle was measured three times, and the averaged value was used as the final result.

*LOD Calculation Procedure and Results: 3σ Method Based on Logarithmic Calibration Curve*

Based on the linear regression curve of logarithmic concentration values and wavelength, we obtained the calibration curve: $Y=3.942*lg(C)+7.837$. We measured 10 blank samples using a matrix sample containing no target analyte (pure buffer solution), and recorded the signal value for each measurement. We calculated the mean $C_{LOD}={10}^{\frac{\bar{Y}_{blank}+3\sigma-b}{k}}$and standard deviation of the blank signals $\sigma=\sqrt{\frac{\sum\left( Y_{i}-\bar{Y}_{blank} \right)^{2}}{n-1}}$, $C_{LOD}\approx0.074$ aM, $\sigma=0.3525$.

The limit of detection was calculated using the 3σ method, where σ represents the standard deviation of blank measurements. A calibration curve was constructed by plotting the signal response $\left( Y \right)$ against the logarithm of target concentrations $\left( lg(C) \right)$ via linear regression, yielding the equation:

$$Y=3.942*lg(C)+7.837$$

where $Y$ is the measured signal, $C$ is the target concentration, and the slope $\left( k \right)$ and intercept $(b)$ were determined as 3.942 and 7.837, respectively. For LOD calculation, 10 blank measurements were performed using pure buffer solution (matrix-matched blank without target analyte). The standard deviation of blank signals was computed as:

$$\sigma=\sqrt{\frac{\sum\left( Y_{i}-\bar{Y}_{blank} \right)^{2}}{n-1}}$$

where $Y_{i}$ is the i-th blank signal. $\bar{Y}_{blank}$ is the mean blank signal, and $\boldsymbol{n=10}$ (number of blank replicates) . The calculated $\sigma$ was 0.3525, with $\bar{Y}_{blank}=2.315$.

The LOD was derived by substituting the critical signal threshold $\left( \bar{Y}_{blank}+3\sigma\right)$ into the calibration curve equation and solving for $C$ :

$$C_{LOD}={10}^{\frac{\bar{Y}_{blank}+3\sigma-b}{k}}$$

The LOD was determined to be 0.074 aM.


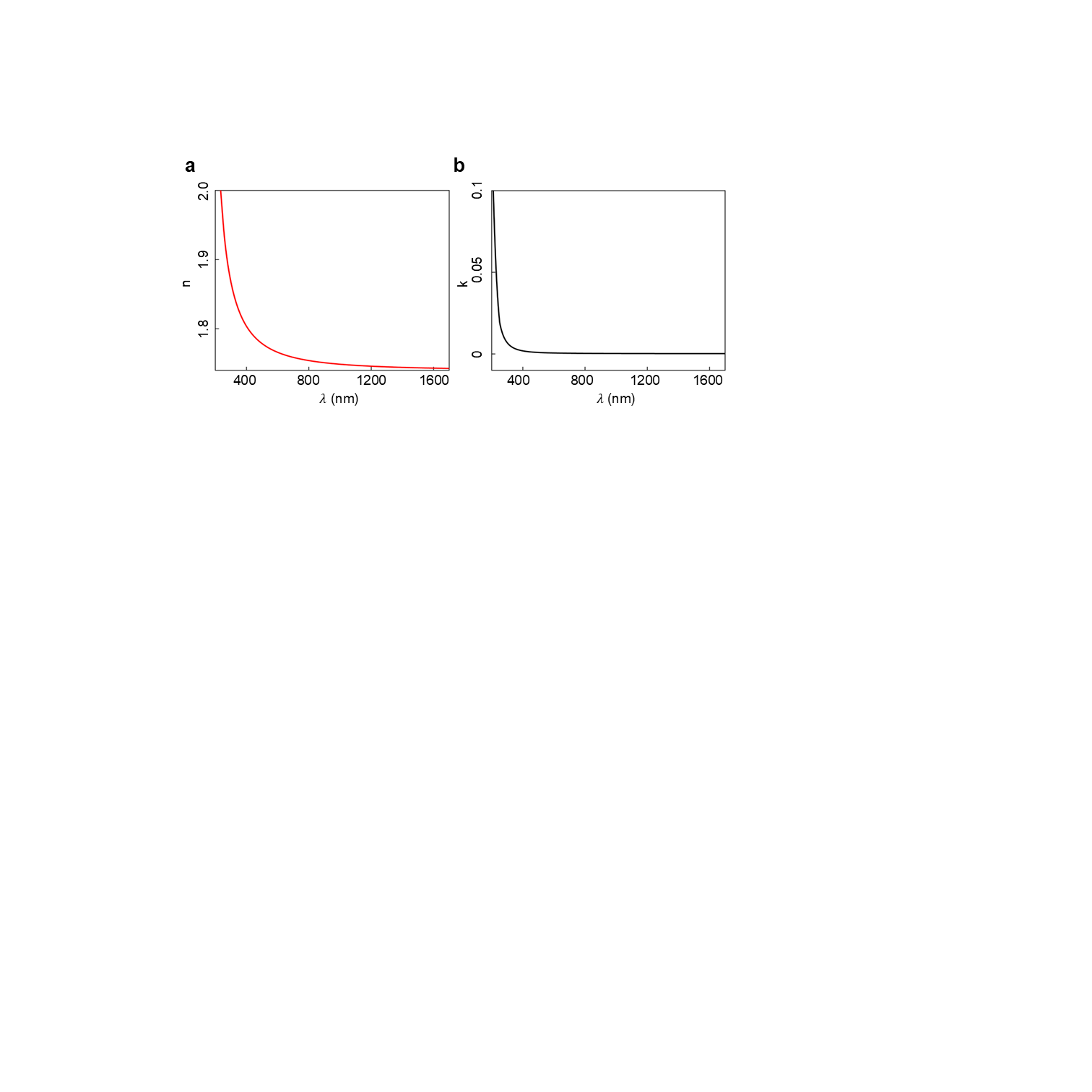
**Figure S6 a** The measured real parts of the refractive index. **b** Imaginary part.

**Table S4** Oligonucleotide sequences employed in this work.

| **Name** | **Sequence (5’-3’)** |
| --- | --- |
| Probe | TAAGCATGATGACCTATG |
| ctDNA (IDH1.R132H) | *TTGTGAGTGGATGGGTAAAACCTA*TCAT**CATAGGTCATCATGCTTA**TGGGGATCAA |
| IDH1 *wt* | *TTGTGAGTGGATGGGTAAAACCTA*TCAT**CATAGGTCGTCATGCTTA**TGGGGATCAA |
| H1 | Biotin-*TAGGTT***TTACCCATCCACTCACAA***CTAGCT***TTGTGAGTGGATGGGTAA** |
| H2 | **TTGTGAGTGGATGGGTAA***AACCTA***TTACCCATCCACTCACAA***AGCTAG*-Biotin |
| Strand 1 | SH-GTCTATAGCCAGTGACGGAGAAGACGAGCAGCGTATACGTAGAATAGTAGGTGGGAGTCGTCCT |
| Strand 2 | SH-CTCCGTCACTGGCTATAGACAATGCTCAGGGATCTCGGTCGCAAATGACTTGCTGCGTACGAGC |
| Strand 3 | CAGCGTTGCTCACCCATACCAACTACGTATACGCTGCTCGTCAAGCTCGTACGCAGCAAGTCATTTTTTTTTTT**TAAGCATGATGACCTATG** |
| Strand 4 | SH-GGTATGGGTGAGCAACGCTGAAGCGACCGAGATCCCTGAGCAAAAGGACGACTCCCACCTACTA |
| Strand 5 | SH-CCTCCTCAGCAAGAGAATGCAATCTCTGCTACGAGGTCTACCAACGACAGCTGGAAGTCCACTT |
| Strand 6 | SH-GCATTCTCTTGCTGAGGAGGAACAGCACTCTAGGAGCATACGAACGTCGTACTGCACCTCTGAA |
| Strand 7 | GGATACAGGTCCTCCAGGTCAAGGTAGACCTCGTAGCAGAGAAATTCAGAGGTGCAGTACGACGTTTTTTTTTT**TAAGCATGATGACCTATG** |
| Strand 8 | SH-GACCTGGAGGACCTGTATCCAACGTATGCTCCTAGAGTGCTGAAAAGTGGACTTCCAGCTGTCG |

| 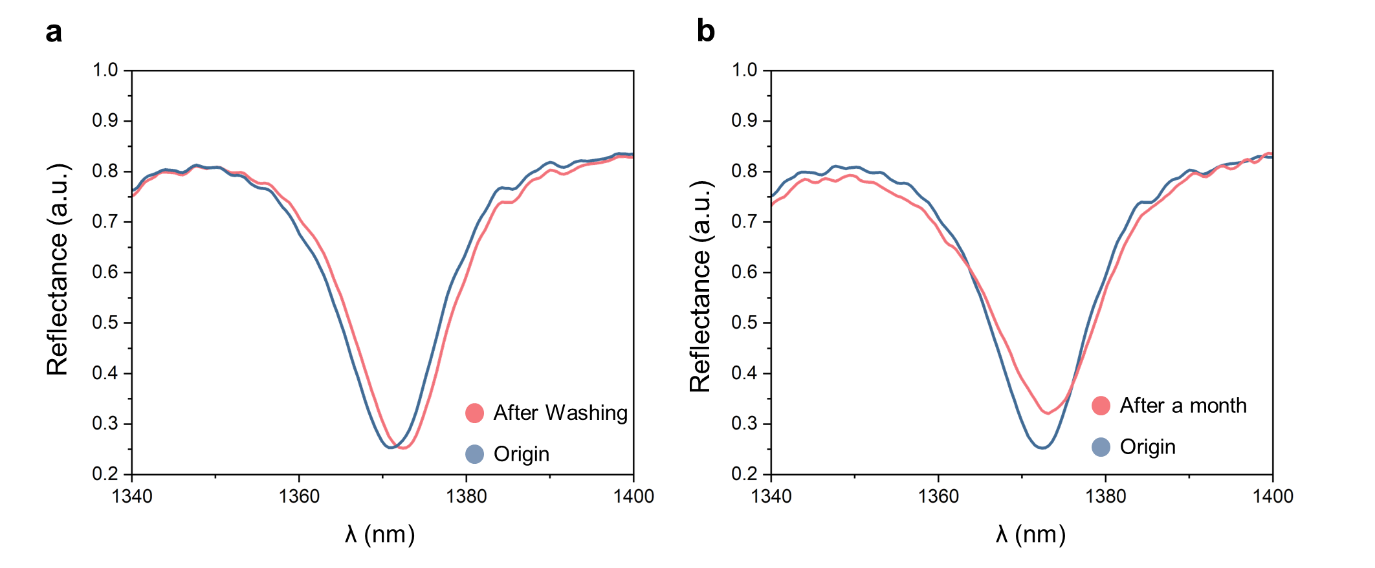 |
| --- |

**Figure S7** Stability evaluation of the metasurface device. **a** Reflection spectra before and after cleaning. **b** Time-varying reflectance spectrum characterization.


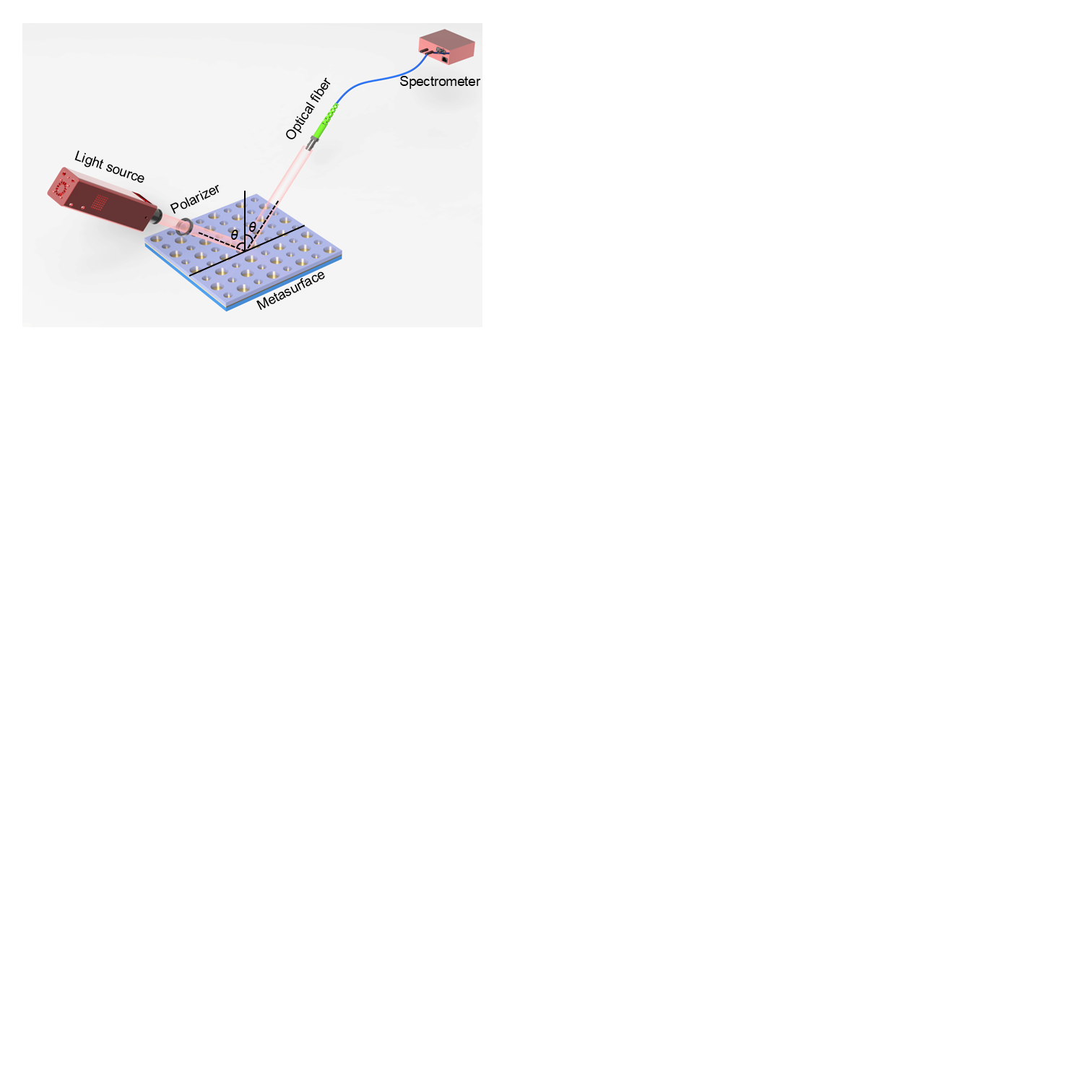


**Figure S8** DCSU-Biosensor testing optical path diagram.
